# Clinical profiles of adverse drug reactions spontaneously reported at a single Korean hospital dedicated to children with complex chronic conditions

**DOI:** 10.1371/journal.pone.0172425

**Published:** 2017-02-15

**Authors:** Bomi Kim, Sunwha Zara Kim, Jin Lee, Ae Hee Jung, Sun-Hoi Jung, Hyeon-Joo Hahn, Hye Ryun Kang, Dong In Suh

**Affiliations:** 1 Department of Pharmacy, Seoul National University Hospital, Seoul, Republic of Korea; 2 Student in training, Bachelor of Medicine and Bachelor of Surgery (MBBS), Shanghai Medical College of Fudan University, Shanghai, China; 3 Regional Drug Safety Monitoring Center, Seoul National University Hospital, Seoul, Republic of Korea; 4 Division of Allergy, Department of Internal Medicine, Seoul National University Hospital, Seoul, Republic of Korea; 5 Department of Pediatrics, Seoul National University Hospital, Seoul, Republic of Korea; National Chiao Tung University College of Biological Science and Technology, TAIWAN

## Abstract

Children with complex chronic conditions (CCC) are presumed to be vulnerable to adverse drug reactions (ADRs). The clinical profiles of ADRs in CCC are not well known. Herein, we aim to describe the ADR profiles in CCC with regard to typical presentations and vulnerable groups. We accessed the ADR yearly reports at a tertiary children's hospital whose practice is mainly dedicated to CCC and descriptively analyzed their clinical profiles according to the presence of a complex chronic condition, ADR severity, and age groups. A total of 1841 cases were analyzed, among which 1258 (68.3%) were mild, 493 (26.8%) moderate, and 90 (4.9%) cases were severe. A total of 1581 (85.9%) cases of complex chronic condition were reported. The proportion of CCC in each severity group increased as the ADR becomes more severe. In CCC, ADRs were most frequently reported by nurses in the adolescent group and in cases where the symptoms involved the gastrointestinal system. The class of antineoplastic and immunomodulating drugs was the most commonly suspected of causing an ADR, followed by one of the antibiotics. When we focus on the trend across the age groups, the ratio of severe-to-total ADRs decreased with older age. Among severe cases, the ratio of off-label prescription-related cases was the highest in the infant/toddler group and decreased as the groups aged. In conclusion, ADRs of CCCs admitted to a tertiary children’s hospital have a unique profile. These groups are vulnerable to ADRs and thus they should be monitored closely, especially when they are infants or toddlers, so that severe ADRs can be identified and treated immediately.

## Introduction

Adverse drug reactions (ADRs) are defined as noxious and unintended responses to a medicine [1]. Persistent ADR monitoring is indicated in medicine since the safety information collected during clinical trials is not sufficient to predict all ADRs that may occur after use of a medication [2]. Particularly, severe ADRs need to be intensively monitored as they can cause permanent harm and even threaten the patient’s life; the need for intensive medical care could potentially be avoided if the ADR is recognized immediately after its occurrence [3]. Hence, clinicians need to be aware of the common types of ADRs and the management of serious ADRs and should be vigilant upon their occurrence.

Pediatric complex chronic conditions are defined as any medical condition that can be reasonably expected to last a minimum of 12 months (unless death intervenes) and to involve either several different organ systems or 1 system severely enough to require specialty pediatric care and often a period of hospitalization in a tertiary care center [4]. Children with complex chronic conditions (CCC) usually have a diversity of conditions and multisystem diseases and require multiple medications, multiple subspecialists, and sometimes off-label prescriptions [5,6]. CCC are thought to be at risk for death and, for the reasons mentioned above, may be vulnerable to ADRs during their hospitalization.

Although many studies have reported ADR profiles in children [7–16], data regarding ADRs in CCC are scarce. In the present study, we assessed the clinical profiles of ADRs reported at a single tertiary children’s hospital whose largest portion of practice is dedicated to CCC by analyzing a database containing archives of ADR cases reported to a regional monitoring center. We tried to recognize the clinical characteristics of ADRs with a special focus on the underlying illnesses, CCCs, severe ADRs, and the most vulnerable subject groups.

## Methods

To determine the clinical profiles of ADRs, we analyzed the yearly data on ADR cases spontaneously reported in the Seoul National University Children’s Hospital (SNUCH) [17]. This institute is the only children’s hospital in Korea that covers not only all divisions of pediatrics but also all pediatric divisions of medicine by dedicated medical staff, which enables the institute to function as the nationwide referral hospital. The ADR surveillance team of the SNUCH was launched in June 2012 as a division of the Seoul National University Hospital and has been reporting pediatric ADR cases to the regional pharmacovigilance center since that time.

In the present study, we accessed a set of de-identified data on ADRs from the 2014 database, recorded from January 1 to December 31, 2014. To set up the working database, we included cases reported by SNUCH and excluded cases reported from the outpatient department. Subjects, even if under the age of 19, treated at the Seoul National University Hospital but not in the SNUCH were excluded from the analysis. Conversely, subjects aged 19–25 years who experienced ADRs during their admission at SNUCH for a rare underlying disease were included in the analysis. All cases of ADRs during hospital admission were included even if they were caused by a drug that was prescribed outside of the hospital, although cases were excluded if detailed information regarding the drug name, time of administration, or dosage was missing. The study protocol was approved by the institutional review board of Seoul National University College of Medicine and Seoul National University Hospital (IRB No. 1508-058-694). Informed consent was waived by the institutional review board.

To interpret the results, we divided the cases according to age. We established the age groups according to the criteria suggested by the Ministry of Food and Drug Safety at the International Conference on Harmonisation [18] as follows: infants/toddlers (28 days to 23 months), preschoolers (2 to 5 years), children (6 to 11 years), adolescents (12 years to 18 years), and adults (≥19 years). Considering that the majority of patients aged ≥19 years are admitted to the adult ward (out of the SNUCH), this age group was excluded from the age-group specific sub-analyses. CCC were verified by applying the updated pediatric complex chronic conditions classification system (CCC v2) to the subjects’ major diagnosis codes in the International Classification of Disease 10th Revision (ICD-10) [19]. Patients’ oncology diseases were verified by obtaining database-matched information on the subjects’ co-payment code from an information service team in the hospital.

Since cases of ADRs are registered through the corresponding menu in the Electronic Medical Records system of the hospital, the patients or caregivers are not allowed to submit ADR reports by themselves; instead, they indirectly report ADRs by providing information to their medical staff, i.e. attending nurses, instructing pharmacists, or physicians. Thus, only the doctors, nurses, and pharmacists constitute the reporting sources.

The type of adverse reaction was classified based on the registered included terms of the World Health Organization (WHO) Adverse Reaction Terminology codes [20] and the suspected drugs were classified based on the Anatomical Therapeutic Chemical (ATC) code [21]. At this point, when multiple suspected drugs and adverse events were observed in a single report, each of them was counted independently. In other words, when a case was listed with two drugs and three symptoms, two cases were included in the drug-specific analysis and three cases were included in the symptom-specific analysis.

The causality was adopted from data registered in the database. Before reporting to the regional monitoring center, trained nurses summarized each adverse event and suggested the causality according to the criteria of the WHO Uppsala Monitoring Center [22]; the suggested causality was confirmed by a pediatrician in charge of the ADR surveillance team at SNUCH. We included all possible, probable, and certain cases, which accounted for the majority of cases, whereas cases of uncertain causality, such as unlikely or unidentifiable cases, were excluded from the analysis.

### Severity assessment

The severity of each ADR was evaluated based on the Hartwig severity scale [3] or the Common Terminology Criteria for Adverse Events (CTCAE) 4.0 [23]. On the Hartwig scale, levels 1 and 2 correspond to mild cases, and levels 3 and 4 to moderate cases. Therefore, ADRs that required treatment, with the suspected drug being held, discontinued, or otherwise changed, ADRs that required an antidote or other treatment, and ADRs that increased the length of hospital stay by at least 1 day were regarded as moderate cases. Severe cases were defined as those with a Hartwig level of 5, 6, or 7, which included any level of ADR that required intensive medical care, ADRs that caused permanent harm to the patient either directly or indirectly, and ADRs that led to the death of the patient. Meanwhile, cases that presented with abnormal lab results were evaluated on the CTCAE severity scale, with grades 1–2 classified as mild, 3 as moderate, and 4–5 as severe cases. When the evaluation standard between the Hartwig scale and CTCAE differed, the higher severity value was used for the subsequent evaluation.

### Off-label prescription

For severe ADR cases, we requested subjects’ identification numbers and verified whether the prescription was in accordance with the label by reviewing medical records retrospectively. After collecting information regarding the clinical indication confirmed by the pediatrician (Dong In Suh), the forms, route of administration (oral/intravenous etc.), dose, and dosage were entered and compared to those listed on the label relevant to the clinical indication. If the prescription met all of the labeled instructions in terms of the indication, age, form, route, and dosage, we classified the case as on-label. If it failed to meet any item on the label, we classified it as off-label. To set the standard for the off-label evaluation, we referred to the criteria provided on the websites of the KFDA [24] and the US-FDA [25]. For the drugs that were suspected to cause severe ADRs, most labels available in both references presented no differences in the indication, age, form, route, and dosage between the two countries; thus, the KFDA criteria were used to evaluate the off-label prescription.

### Statistics

Statistical analysis was conducted by using SPSS Statistics 23.0 (SPSS, Chicago, Illinois, USA). Categorical data were analyzed by the chi-square test or the Fisher’s exact test, and the tendency of the ratios (ratios of severe-to-total ADRs and off-label-to-total prescription) by age groups was tested by the chi-square test for trend. The significance level was set at *p*<0.05.

## Results

### Subjects being analyzed

**Fig 1:**
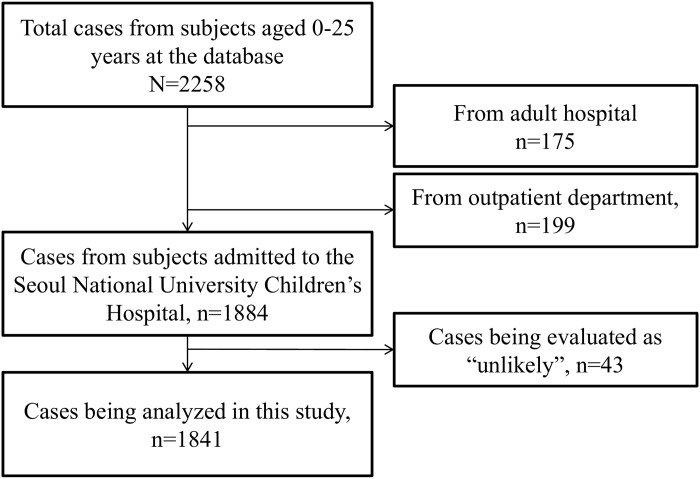
Flowchart of the subjects being analyzed.

A flowchart of the reported cases enrolled in this analysis was shown in Fig 1. A total of 1841 cases (889 patients, including 2657 drug suspects and 2380 reactions) met all inclusion/exclusion criteria and were included in the analysis. In terms of causality, 13 cases were classified as definite, 399 cases were probable, and the other 1429 cases were classified as possible. Their severity profiles according to sex and disease group are presented in Table 1. A total of 1258 (68.3%), 493 (26.8%), and 90 (4.9%) cases out of 1841 total cases were classified as mild, moderate, and severe, respectively. While 1090 (59.2%) cases happened in males, 751 (40.8%) cases occurred in females. In CCC, 1581 (85.9%) cases were reported, among them 1057 were mild, 443 moderate, and 81 were severe ADRs. The proportion of CCC in each severity group increased with increasing ADR severity (P for trend = 0.002).

**Table 1:** The number of ADR reports by sex, underlying disease group, and severity.

Characteristics	No. of ADR Reports				*P* value^+^
	Total	Mild	Moderate	Severe	
**Sex**					
Male	1090	743	292	55	
Female	751	515	201	35	
Total	1841	1258	493	90	
Proportion of males (%)	68.9	59.1	59.2	61.1	0.706
**Underlying disease group**					
Chronic complex condition	1581	1057	443	81	
Other diseases	260	201	50	9	
Total	1841	1258	493	90	
Proportion of CCCs (%)	85.9	84.0	89.9	90.0	0.002*

ADR, adverse drug reaction; CCC, children with chronic complex condition.

^+^Chi-square test for trend

*Statistically significant

### Reporting routes

Table 2 shows the ADR profiles according to the reporting route. Most ADR cases were reported by nurses, both in total subjects (89.6%) and in the CCC (88.5%) groups, followed by doctors and pharmacists. Nurses also reported most often in severe cases; however, the ratio of severe-to-total cases in each reporter group was the highest in pharmacists both in total subjects (12.9%) and in CCC (13.8%).

**Table 2:** Cases according to the reporting sources.

Type of Reporter	Total subjects			Children with complex chronic conditions		
	ADR reports, n (%)	Severe ADR reports, n	Severe/Total ADRs, %	ADR reports, n (%)	Severe ADR reports, n	Severe/Total ADRs, %
Nurses	1649 (89.6)	73	4.4	1399 (88.5)	65	4.6
Physicians	107 (5.8)	6	5.6	102 (6.5)	5	4.9
Pharmacists	85 (4.6)	11	12.9	80 (5.1)	11	13.8
Total	1841 (100)	90		1581 (100)	81	

ADR, adverse drug reaction.

### Drugs

In terms of the suspected drugs, a total of 2657 drugs were related to ADRs, which is greater than the actual number of ADR reports. This is because we independently counted each drug as a separate drug-ADR pair when there were multiple suspected drugs in a single report. A total of 1770 (66.6%), 725 (27.3%), and 162 (6.1%) drugs were related to mild, moderate, and severe ADRs, respectively. Regarding CCC, 2353 drugs were associated with ADRs, of which 1549 (65.8%), 660 (28.0%), and 144 drugs (6.1%) were associated with mild, moderate, and severe ADRs, respectively.

Table 3 lists the ATC class distribution of the drugs, and Table 4 lists the top 10 drugs causing ADR events in CCC. The most frequently suspected ATC class to cause ADRs was the antineoplastic and immunomodulating agents (ATC class L, comprising a total of 846 cases with 723 cancer chemotherapeutics and 123 immunosuppressive agents) accounting for 36.0% of all ADRs. This was closely followed by systemic antiinfectives (651 cases, 27.7%), nervous system drugs (396 cases, 16.8%), and cardiovascular system drugs (161 cases, 6.8%) (Table 3). The top 10 most frequent drug suspects were antineoplastic agents (5 drugs), followed by systemic antiinfectives (3 drugs). The single most frequent drug suspect was fentanyl (190 cases) followed by piperacillin/tazobactam (123 cases). Among the antineoplastic agents, etoposide (112 cases, 13.3%) was most frequently associated with ADRs followed by carboplatin (79 cases) and cyclophosphamide (58 cases). More detailed information is shown in S1 File.

**Table 3:** ATC classes distribution causing ADR events in CCC.

ATC class	Events (%)
[L] Antineoplastic and immunomodulating agents	846 (36.0)
[J] Antiinfectives for systemic use	651 (27.7)
[N] Nervous system	396 (16.8)
[C] Cardiovascular system	161 (6.8)
[H] Systemic hormonal preparations, excluding sex hormones and insulins	70 (3.0)
[A] Alimentary tract and metabolism	59 (2.5)
[V] Various	57 (2.4)
[B] Blood and blood forming organs	41 (1.7)
[M] Musculo-skeletal system	30 (1.3)
[R] Respiratory system	25 (1.1)
[G] Genito-urinary system and sex hormones	10 (0.4)
[P] Antiparasitic products, insecticides, and repellents	4 (0.2)
[S] Sensory organs	1 (0.0)
Others—Extemporaneous preparations	2 (0.1)
Total	2353 (100.0)

ATC, Anatomical Therapeutic Chemical; ADR, adverse drug reaction; CCC, children with complex chronic conditions.

**Table 4:** Top 10 drugs most frequently causing ADR events in CCC.

ATC class	Drug Name	Events
N	Fentanyl	190
J	Piperacillin/Tazobactam	123
L	Etoposide	112
L	Carboplatin	79
C	Furosemide	68
J	Teicoplanin	66
L	Cyclophosphamide	58
L	Fludarabine	57
J	Meropenem	57
L	Busulfan	56

ADR, adverse drug reaction; ATC, Anatomical Therapeutic Chemical; CCC, children with complex chronic conditions.

### ADR distribution according to system-organ class

In terms of adverse reactions, a total of 2380 reactions were recorded when we independently counted each adverse reaction as a separate event. In CCC, 2076 ADRs were recorded. Since each ADR can be classified as affecting up to 3 system organ classes as suggested by the WHO-Adverse Reaction Terminology, ADRs were summed up into 3350 and 2917 system-organ classes (SOCs) from total subjects and CCC, respectively.

Table 5 shows the frequency distribution of SOCs and Table 6 shows the top 10 most prevalent reactions manifested as ADR events in CCC. Gastrointestinal system disorders were the most prevalent (851/2917 cases, 29.2%) followed by autonomic nervous system disorders (537 cases, 18.4%), body as a whole general disorders (326 cases, 11.2%), and skin and appendages disorders (242 cases, 8.3%) (Table 5). In terms of each single ADR, nausea was the most common reaction (322/2076 events, 15.2%) followed by vomiting (247 events), diarrhea (161 events), and abnormal hepatic function (76 events) (Table 6). More detailed information is available in S2 File. Some ADRs (i.e., vomiting, diarrhea, and abdominal pain) ranked high in more than one organ system.

**Table 5:** Frequency distribution of ADR events according to systemic-organ classes (SOCs) in CCC.

System-Organ Class	Total ADRs (%)	
Gastro-intestinal system disorders (0600)	851	(29.2)
Autonomic nervous system disorders (0420)	537	(18.4)
Body as a whole general disorders (1810)	326	(11.2)
Skin and appendages disorders (0100)	242	(8.3)
Central and peripheral nervous system disorders (0410)	191	(6.5)
Liver and biliary system disorders (0700)	160	(5.5)
Metabolic and nutritional disorders (0800)	130	(4.5)
Urinary system disorders (1300)	107	(3.7)
General cardiovascular disorders (1010)	63	(2.2)
Heart rate and rhythm disorders (1030)	49	(1.7)
Psychiatric disorders (0500)	41	(1.4)
Platelet, bleeding and clotting disorders (1230)	34	(1.2)
Musculo-skeletal system disorders (0200)	31	(1.1)
Respiratory system disorders (1100)	28	(1.0)
Red blood cell disorders (1210)	28	(1.0)
White blood cell and reticulo-endothelial system disorders (1220)	23	(0.8)
Application site disorders (1820)	20	(0.7)
Endocrine disorders (0900)	17	(0.6)
Vision disorders (0431)	12	(0.4)
Hearing and vestibular disorders (0432)	10	(0.3)
Vascular (extracardiac) disorders (1040)	6	(0.2)
Resistance mechanism disorders (1830)	6	(0.2)
Reproductive disorders, female (1420)	2	(0.1)
Special senses other, disorders (0433)	1	(0.0)
Reproductive disorders, male (1410)	1	(0.0)
Neoplasms (1700)	1	(0.0)
**Total**	**2917**	**(100.0)**

ADR, adverse drug reaction; CCC, children with complex chronic conditions.

**Table 6:** Top 10 reactions most frequently manifested as ADR events in CCC.

Adverse reactions in preferred terms	Events
Nausea	322
Vomiting	247
Diarrhoea	161
Hepatic function abnormal	76
Rash	75
Abdominal pain	68
Headache	67
Dizziness	65
Fever	63
Hypokalaemia	61

ADR, adverse drug reaction; CCC, children with complex chronic conditions.

### Person-based analyses

A distribution of subject numbers according to events-per-person groups is listed in Table 7. A total of 889 subjects (673 CCCs and 216 non-CCCs) experienced ADRs from once to 22 times during the 1-year period. Whilst the number of ADRs experienced by subjects in the CCC group varied widely (from 1 to 22 events), no subject in the non-CCC group experienced more than 3 events during the 1-year period. When we confined the analyses to severe ADRs, 70 subjects in the CCC group experienced a total of 81 severe ADRs whereas only 9 subjects in the non-CCC group experienced 9 severe ADRs. The proportion of subjects who experienced severe ADRs was higher in the CCC group (70 out of 673 subjects) than the non-CCC group (9 out of 216 subjects) (Fisher’s exact test, p = 0.004). In the CCC group, as the number of events-per-person increased the proportion of subjects who experienced severe ADR events also increased (P for trend <0.001).

**Table 7:** Distribution of persons according to events-per-person, presence of CCC, and their experience of severe ADR.

	Children with chronic complex condition				Children other than chronic complex condition			
Events per person	Number of total subjects	Number of subjects who have experienced severe ADR	Number of subjects who have not experienced severe ADR	Proportion of subjects who experienced severe ADR events‡	Number of total subjects	Number of subjects who have experienced severe ADR	Number of subjects who have not experienced severe ADR	Proportion of subjects who experienced severe ADR events
1	430	14	416	3%	173	4	169	2%
2–3	142	14	128	10%	43	5	38	12%
4–6	44	12	32	27%	0	n/a	n/a	n/a
7–10	36	19	17	53%	0	n/a	n/a	n/a
11–15	12	6	6	50%	0	n/a	n/a	n/a
16–22	9	5	4	56%	0	n/a	n/a	n/a
Total†	673	70	603	10%	216	9	207	4%

^†^Fisher’s exact test, p = 0.004;

^‡^p for trend, p<0.001

### Profiles of pediatric oncology patients

A total of 839 ADRs occurred in oncology patients, which comprised 51% of all CCC cases. In 466 cases, 723 cancer chemotherapeutics were suspected but in the other 373 cases, medicines other than cancer chemotherapeutics (i.e., piperacillin/tazobactam, furosemide, tacrolimus, and so forth) were suspected of causing the ADR. Among the 466 chemotherapeutics-related cases, one single chemotherapeutic was suspected to cause the ADR in 278 cases. On the other hand, a combination of two, three, and four cancer chemotherapeutics were suspected in 122, 63, and 3 cases, respectively.

### Signal detections

Table 8 presents a list of drug-ADR combinations (and their reporting odds ratio) that should be focused as signals in CCC. A total of 18 drug-ADR combinations were calculated as having a lower margin of 95% confidence interval of reporting odds ratio (ROR) larger than 1. The combination of ibuprofen/arginine and laryngitis and that of atomoxetine and weight increase had the highest reporting odds ratio, followed by a combination of heparin and paraesthesia.

**Table 8:** A list of drug-ADR combinations that should be suspected as signals in CCC.

Drug	ADR	Case numbers (n)				Reporting Odds Ratio (ROR)	Lower margin of 95% CI
		suspected drug- suspected ADR	suspected drug- other ADR	other drug- suspected ADR	other drug- other ADR		
Ibuprofen/Arginine	laryngitis	1	2	1	1577	788.5	35.542
Atomoxetine	weight increase	1	1	2	1577	788.5	35.542
Heparin	paraesthesia	1	1	8	1571	196.4	11.273
Lorazepam	hiccup	1	3	5	1572	104.8	9.249
Clofarabine	conjunctival haemorrhage	1	11	1	1568	142.5	8.374
Clofarabine	vision abnormal	1	11	1	1568	142.5	8.374
Cefotaxime	myalgia	1	4	6	1570	65.4	6.343
Joulie`s solution	rash erythematous	1	1	15	1564	104.3	6.227
Oxybutynin	bilirubinaemia	1	1	22	1557	70.8	4.288
Hydrocortisone	dysaesthesia	1	8	5	1567	39.2	4.102
L- Asparaginase	hypotrichosis	2	14	14	1551	15.8	3.285
Potassium chloride	injection site reaction	1	3	16	1561	32.5	3.208
Clonidine	speech disorder	1	27	2	1551	28.7	2.527
Vancomycin	laryngitis	1	52	1	1527	29.4	1.812
Anti thymocyte globulin	conjunctivitis	1	40	2	1538	19.2	1.708
Heparin	dizziness	1	1	64	1515	23.7	1.464
Vancomycin	dystonia	1	52	2	1526	14.7	1.310
Propacetamol	injection site reaction	1	9	16	1555	10.8	1.291

ADR, adverse drug reaction

### Age groups

**Fig 2:**
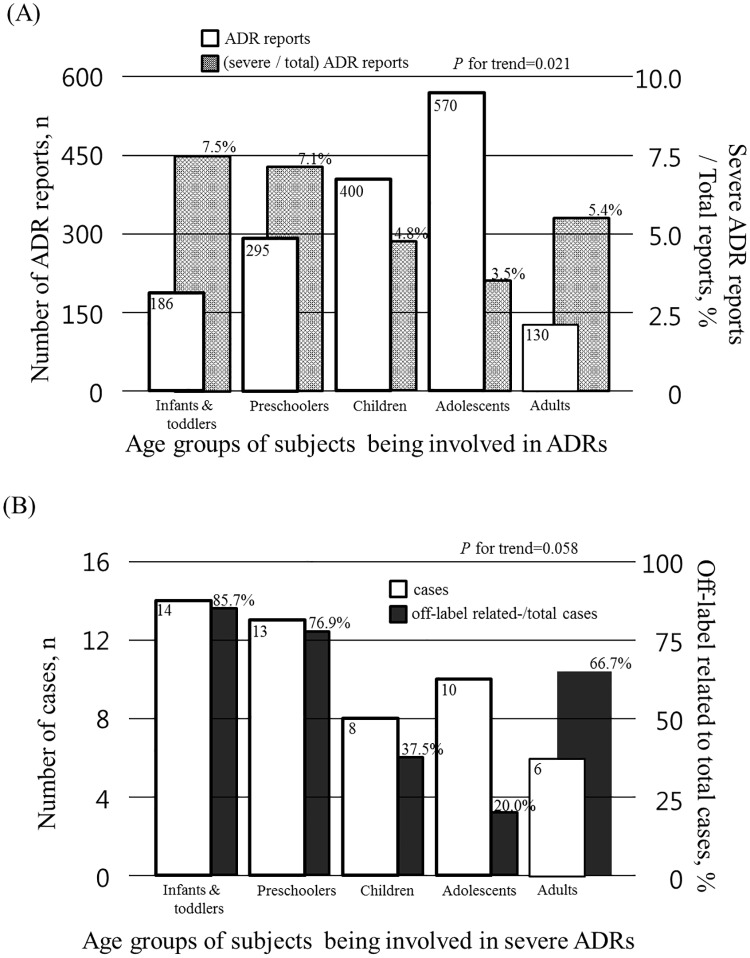
ADR profiles across the age groups. (A) The number of ADR reports and the ratio of severe-to-total ADR reports across the age groups in children with CCCs. Numbers are presented as empty bars and the ratios are presented as solid bars. (B) The number of cases and their ratio of off-label prescription in the 51 severe ADR cases in CCCs. Numbers are presented as empty bars and the ratios are presented as solid bars. ADR, adverse drug reaction; CCC, children with complex chronic condition.

The distribution of the number of ADR reports according to the severity and age group is displayed in Table 9 and Fig 2. A total of 635 (34.5%) cases were reported in the adolescent group, which was the highest among the age groups. The groups of children (490 cases, 26.6%), preschoolers (346 cases, 18.8%), infants/toddlers (229 cases, 12.4%) and adults (141 cases, 7.7%) followed consecutively. The ratio of severe-to-total ADRs decreased with increasing age (P for trend = 0.013, Table 9).

**Table 9:** Number of ADR reports according to severity and age group.

**In all subjects**						
	Infants/toddlers	Preschoolers	Children	Adolescents	Adults	Total
Mild or moderate	212	324	467	614	134	1751
Severe	17	22	23	21	7	90
Total	229	346	490	635	141	1841
Severe/total	7.4	6.4	4.7	3.3	5.0	P for trend = 0.013
**In children with complex chronic condition**						
	Infants/toddlers	Preschoolers	Children	Adolescents	Adults	Total
Mild or moderate	172	274	381	550	123	1500
Severe	14	21	19	20	7	81
Total	186	295	400	570	130	1581
Severe/total	7.5	7.1	4.8	3.5	5.4	P for trend = 0.021
**In children without complex chronic condition**						
	Infants/toddlers	Preschoolers	Children	Adolescents	Adults	Total
Mild or moderate	40	50	86	64	11	251
Severe	3	1	4	1	0	9
Total	43	51	90	65	11	260
Severe to total	7.0	2.0	4.4	1.5	0.0	P for trend = 0.193

In CCC, the number of ADR reports and the ratio of severe-to-total ADRs across the age groups are shown in Table 2 and Fig 2A. Except for the adult group, there was a similar trend of increasing number of ADRs with increasing age; however, the ratio of severe-to-total ADRs decreased with increasing age (P for trend = 0.021). These trends were not observed in non-CCC subjects (P for trend = 0.193).

### Off-label use and the severe ADR

Among the 90 cases of severe ADRs in the total population, 81 cases of severe ADRs occurred in CCC and they were associated with a total of 144 prescriptions. The number of subjects or prescriptions including off-label prescriptions according to the cancer chemotherapeutics or others is shown in Table 10. Since cancer chemotherapeutics are prescribed based on standardized protocols, we excluded these 60 cancer chemotherapeutic prescriptions from analyses. The medical records concerning the 84 remaining prescriptions of non-cancer medications for 51 cases were analyzed according to the KFDA criteria. A total of 29/84 (34.5%) prescriptions were off-label. Fig 2B shows the number of cases and their ratio of off-label prescription across all age groups in the 51 severe ADR cases in CCC. A total of 29 cases (34.5%) were classified as off-label. The infant/toddler group ranked highest among the CCC group with severe ADRs (14/51 cases, 27.5%) followed by the preschooler group (25.5%). The percentage of off-label prescription-related cases was the highest in the infant/toddler group and tended to decrease with patient age (P for trend = 0.058) (Fig 2B). When we excluded the adult group (because it did not represent the whole adult CCC group), the tendency was more significant (P for trend = 0.004).

**Table 10:** Number of subjects or prescriptions, including off-label prescriptions, according to cancer chemotherapeutic or other medication in children with complex chronic condition.

		Prescription type			Medication other than cancer therapeutics, total† [B]+[C]
		Cancer chemotherapeutics only [A]	Cancer chemotherapeutics with other medication [B]	Other medication only [C]	
**Infant/toddlers**	**Subjects**	**0**	**0**	**14**	**14**
	**Total prescriptions**			**16**	** **
	**Off-label prescriptions**			**10**	**10**
**Preschoolers**	**Subjects**	**8**	**2**	**11**	**13**
	**Total prescriptions**	**(17)**	**(2)+4**	**16**	** **
	**Off-label prescriptions**		**1**	**9**	**10**
**Children**	**Subjects**	**11**	**1**	**7**	**8**
	**Total prescriptions**	**(18)**	**(1)+1**	**11**	** **
	**Off-label prescriptions**			**3**	**3**
**Adolescents**	**Subjects**	**10**	**0**	**10**	**10**
	**Total prescriptions**	**(15)**		**21**	** **
	**Off-label prescriptions**			**2**	**2**
**Adults**	**Subjects**	**1**	**2**	**4**	**6**
	**Total prescriptions**	**(5)**	**(2)+6**	**9**	** **
	**Off-label prescriptions**		**1**	**3**	**4**
**Total**	**Subjects**	**30**	**5**	**46**	**51**
	**Total prescriptions**	**(55)**	**(5)+11**	**73**	
	**Off-label prescriptions**		**2**	**27**	**29**

^†^P for trend = 0.058

Numbers in parentheses indicates the number of chemotherapeutic agents.

## Discussion

In this study, we analyzed the profiles of ADRs reported in a nationwide referral children’s hospital that treats a large proportion of CCC. More than four-fifths of ADRs were reported in CCC, among them pediatric oncology cases comprised the majority. The ratios of severe-to-total ADRs were higher in CCC. In further analysis of cases of ADRs in CCC, younger groups tended to have higher severe-to-total ADRs and have a greater chance of being exposed to off-label prescription. Although the study was conducted on admitted patients in a single institute, this is the first study to describe the profiles of ADRs in a specialized population of children largely comprised of CCC.

Being spontaneously reported, the ADR profile is largely affected by the patient composition and by the practice pattern. The proportion of CCC among the in-hospital patients is exceptionally high in the authors’ hospital compared to ones in the other general children’s hospitals. The authors’ institution functions as the final level of referral in Korea where children are finally transferred and treated when they have a very rare disease, multidisciplinary and complex problems, serious complications, or refractory responses despite current standard managements. Therefore, the collected data may provide a valuable clue for understanding ADR profiles accompanying the practice for CCC.

As a single center report, the number of yearly cases is relatively high compared to the previous ones from other single institutes [10,12,26]. In CCC, ADRs are most frequently reported in the adolescent group (34.5%) and most commonly involved the gastrointestinal system (35.1%); these results are comparable to those of previous studies. In adults, previous studies have shown that older age groups tend to have many ADRs [27,28], although the meaning of older age in adults differs from that in children. In children, however, the most frequent age groups were so varied across all studies that we could not conclude any trend solely from the number reports [7,10,12,29]. Regarding the affected organ system, several previous studies reported that the gastrointestinal system and skin were frequently involved [7,10,12,14,16,29–31], whereas in other studies the central and peripheral nervous systems were also frequently affected [7,10,14,29]. In adults, more organs were involved according to the clinical characteristics of the subjects enrolled [26–28,31–35]. After excluding antineoplastic agents, antibiotics ranked the first among the suspected drugs in our study, followed by systemic nervous system and cardiovascular drugs, which was similar to in previous reports [7,10,29–31]. These results indicate that ADRs in CCC have a unique profile compared to ADRs adult patients [26,28,33,35,36] or other children [16,29].

Since severe ADRs may cause irreversible damage [3], even if rare, we need to be aware of them. To verify whether CCC are a more vulnerable group to severe ADRs, we evaluated the association of ADR severity with the proportion of CCC in the total subjects and found an increasing tendency as the ADR became more severe. In further analyses confined to CCC cases, the proportion of severe-to-total ADRs was the highest in the infant/toddler group and then decreased with older age. This is comparable to previous findings that reported that the ratio of severe ADRs was high in 0- to 1-year-old subjects [12,16]. Even though the present study was limited due to the lack of information regarding the total prescription of all drugs as well as the occurrence ratio of all ADRs, the above results suggest that we should observe and monitor for potential ADRs intensively when using antibiotics or nervous system drugs in CCC, especially in younger ones.

There is no clear explanation as to why ADRs happen more often and are more severe in children with CCCs. Based on the chronic and complex nature of our subjects’ condition, we could speculate that several reasons exist with regard to the children themselves or the drugs that are administered to those children. In contrast to general subjects, CCC is less stable so they might have less in reserve to maintain homeostasis and thus they may require concomitant multiple drug administration. As for antibiotics, piperacillin/tazobactam, teicoplanin, and meropenem, which are used primarily for severe infections, ranked the highest in this study. Conversely, there were very few ADRs from amoxicillin or cephalosporin, which are frequently used in the treatment of community-acquired infections. Moreover, an off-label prescription may have affected the trend. Considering that there may be insufficient data on medication regarding the quality, efficacy, and safety in young CCCs from clinical trials [37], there might be a greater chance of medicine being prescribed in an off-label manner. In a further analysis of ADR cases in CCC, the younger age groups tended to have a higher ratio of severe-to-total ADR and subjects who had severe ADRs had a greater chance of being exposed to off-label prescriptions. Furthermore, chronic exposure to several drugs may cause a change in the receptor profiles via the physiological feedback loop. Lastly, in oncology patients, the dosage of chemotherapeutics administered is the maximum tolerable dose, which can damage hepatic or renal function which is essential for drug metabolism. On the other hand, these findings can merely be a form of survivor bias. CCC stemming from congenital problems die early when they have detrimental natural courses; therefore, the older children selectively survive when they have milder illnesses.

One of the interesting features of reports of ADRs in this institution was that the majority were made by nurses, which is in line with other reports [7,27] and may be related to the policy in the authors’ institution of encouraging reporting activity mainly through the nursing system. We assumed that the nurses encounter more ADRs due to their inherent job characteristics through which they receive the patients’ claims and access medical records while administering drugs to the patient according to the attending doctor’s instruction. Such a system based on nurses has the virtue of registering more cases, with a minimal case loss, compared to a system based on the doctors or caregivers as the primary reporting source [38,39]. However, this also has an inherent risk of deviating ADR profiles into subjective symptom-based ones rather than the laboratory change-based ones in the presymptomatic phase. Moreover, compared to the other groups of medical personnel, pharmacists rarely reported ADRs, whereas their ratio of severe-to-total ADRs was the highest in this study, which may be related to their role in clinical practice. Instead of contacting the patient in person, pharmacists review the prescription and, in some cases, the medical records of the patients. Indeed, most reports from pharmacists were made during their retrospective review of the medical records by finding omitted ADR cases. Considering that they detect, assess, and cope with any rare but potentially relevant ADRs, this result shows how important the role of the pharmacist is in the monitoring of ADRs.

We acknowledge that there were a number of limitations of our study and that caution should be taken when generalizing our findings. First, this was a retrospective study, which did not review all prescription records of the entire pediatric population. We analyzed spontaneous reports, resulting in an inherent risk of missing some of the under-recognized ADRs, and it is possible that these missing events may have disproportionately occurred due to a certain subset of drugs [40]. Therefore, despite the fact that the low numbers of cases with the respective event-drug-pairs (n<3) do not allow finding signals, drug-ADR combinations listed in the Table 8 need to be verified in further large-scale pharmacovigilance studies. Lastly, we could not verify the role of polypharmacy on severe ADRs due to lack of information on the whole drugs concomitantly administered at the time of ADR event. Nonetheless, the results of our study imply that vulnerable patient groups exist in terms of the more frequent and/or severe ADRs and emphasize that more vigilant monitoring is required when specific drugs are prescribed to CCC, especially younger ones.

In conclusion, ADRs of CCC admitted to a tertiary children’s hospital show a unique profile compared to those reported in adults or children in other clinical settings. In cases of severe ADRs, these are more frequently observed in vulnerable groups such as CCCs and younger children, especially in the infants/toddler group. In order to confirm the effects of off-label prescription on the ADR frequency and severity, a large, prospective study is needed in the future.

## Supporting information

S1 File Frequency distribution of drugs causing ADR events. (XLSX)

S2 File ADRs_distribution_according_to_SOCs_in_CCC. (XLSX)

## References

[1] European Medicines Agency and Heads of Medicines Agencies. Guideline on good pharmacovigilance practices: Annex I—Definitions (Rev 3) [Internet]. 2014 [cited 8 Jan 2016] pp. 1–6. http://www.ema.europa.eu/ema/index.jsp?curl=pages/regulation/document_listing/document_listing_000345.jsp

[2] Smith PB, Benjamin DK, Murphy MD, Johann-Liang R, Iyasu S, Gould B, et al Safety monitoring of drugs receiving pediatric marketing exclusivity. Pediatrics. 2008;122: e628–e633. 10.1542/peds.2008-0585 18762496 PMC2561901

[3] Hartwig SC, Siegel J, Schneider PJ. Preventability and severity assessment in reporting adverse drug reactions. Am J Hosp Pharm. 1992;49: 2229–32. Available from: http://www.ncbi.nlm.nih.gov/pubmed/1524068 1524068

[4] Feudtner C, Christakis DA, Connell FA. Pediatric deaths attributable to complex chronic conditions: a population-based study of Washington State, 1980–1997. Pediatrics. 2000;106: 205–9. Available from: http://www.ncbi.nlm.nih.gov/pubmed/10888693 10888693

[5] Simon TD, Berry J, Feudtner C, Stone BL, Sheng X, Bratton SL, et al Children with complex chronic conditions in inpatient hospital settings in the United States. Pediatrics. 2010;126: 647–55. 10.1542/peds.2009-3266 20855394 PMC2962571

[6] Srivastava R, Stone BL, Murphy NA. Hospitalist care of the medically complex child. Pediatr Clin North Am. 2005;52: 1165–87, x 10.1016/j.pcl.2005.03.007 16009262

[7] Rosli R, Ming LC, Abd Aziz N, Manan MM. A Retrospective Analysis of Spontaneous Adverse Drug Reactions Reports Relating to Paediatric Patients. PLoS One. 2016 6 1;11(6):e0155385 10.1371/journal.pone.0155385 27249414 PMC4889073

[8] Bellis JR, Kirkham JJ, Nunn AJ, Pirmohamed M. Adverse drug reactions and off-label and unlicensed medicines in children: a prospective cohort study of unplanned admissions to a paediatric hospital. Br J Clin Pharmacol. 2014;77: 545–53. 10.1111/bcp.12222 23919928 PMC4371534

[9] Turner S, Nunn AJ, Fielding K, Choonara I. Adverse drug reactions to unlicensed and off-label drugs on paediatric wards: a prospective study. Acta Paediatr. 1999;88: 965–968. 10519338 10.1080/08035259950168469

[10] Digra KK, Pandita A, Saini GS, Bharti R. Pattern of Adverse Drug Reactions in Children Attending the Department of Pediatrics in a Tertiary Care Center: A Prospective Observational Study. Clin Med Insights Pediatr. 2015;9: 73–8. 10.4137/CMPed.S29493 26309424 PMC4533849

[11] Gholami K, Babaie F, Shalviri G, Javadi MR, Faghihi T. Pediatric hospital admission due to adverse drug reactions: Report from a tertiary center. J Res Pharm Pract. 2015;4: 212–215. 10.4103/2279-042X.167045 26645028 PMC4645134

[12] Jung JH, Tae J, Rhew KY. Assessment of Pediatric Adverse Drug Reaction Reports. J Kor Soc Heal Pharm. 2013;30: 108–118.

[13] Thiesen S, Conroy EJ, Bellis JR, Bracken LE, Mannix HL, Bird KA, et al Incidence, characteristics and risk factors of adverse drug reactions in hospitalized children ? a prospective observational cohort study of 6,601 admissions. BMC Med. BMC Medicine; 2013;11: 237 10.1186/1741-7015-11-237 24228998 PMC4225679

[14] Ufer M, Kimland E, Bergman U. Adverse drug reactions and off-label prescribing for paediatric outpatients: A one-year survey of spontaneous reports in Sweden. Pharmacoepidemiol Drug Saf. 2004;13: 147–152. 10.1002/pds.858 15072113

[15] Wallerstedt SM, Brunlof G, Sundstrom A. Rates of spontaneous reports of adverse drug reactions for drugs reported in children: A cross-sectional study with data from the swedish adverse drug reaction database and the swedish prescribed drug register. Drug Saf. 2011;34: 669–682. 10.2165/11591730-000000000-00000 21751827

[16] Li H, Guo X-J, Ye X-F, Jiang H, Du W-M, Xu J-F, et al Adverse drug reactions of spontaneous reports in shanghai pediatric population. PLoS One. 2014;9: e89829 10.1371/journal.pone.0089829 24587066 PMC3933652

[17] Baek HJ, Cho YS, Kim KS, Lee J, Kang HR, Suh DI. Multidisciplinary approach to improve spontaneous ADR reporting in the pediatric outpatient setting: a single-institute experience in Korea. Springerplus. Springer International Publishing; 2016;5: 1435. 10.1186/s40064-016-3151-z PMC5005223 27652011

[18] International Conference on Harmonisation (ICH). Clinical Investigation of Medicinal Products in the Pediatric Population (E11) [Internet]. 2000 pp. 1–16. http://www.ich.org/products/guidelines/efficacy/efficacy-single/article/clinical-investigation-of-medicinal-products-in-the-pediatric-population.html 12362934

[19] Feudtner C, Feinstein JA, Zhong W, Hall M, Dai D. Pediatric complex chronic conditions classification system version 2: updated for ICD-10 and complex medical technology dependence and transplantation. BMC Pediatr. 2014;14: 199 10.1186/1471-2431-14-199 25102958 PMC4134331

[20] World Health Organization the UMC. WHO-ART—WHO Adverse Reaction Terminology [Internet]. [cited 1 Dec 2015].http://www.umc-products.com/DynPage.aspx?id=73589&mn1=1107&mn2=1664

[21] World Health Organization Collaborating Centre for Drug Statics Metholodgy. ATC/DDD Index 2015 [Internet]. [cited 16 Nov 2015]. http://www.whocc.no/atc_ddd_index/

[22] World Health Organization the UMC. The use of the WHO-UMC system for standardised case causality assessment [Internet]. [cited 16 Nov 2015]. http://www.who-umc.org/Graphics/26649.pdf

[23] National Cancer Institute. Common Terminology Criteria for Adverse Events (CTCAE) Common Terminology Criteria for Adverse Events v4.0 (CTCAE) [Internet]. 2009 [cited 7 Jan 2016]. http://evs.nci.nih.gov/ftp1/CTCAE/CTCAE_4.03_2010-06-14_QuickReference_8.5x11.pdf

[24] Korean Ministry of Food and Drug Safety. No Title [Internet]. [cited 8 Jan 2016]. http://ezdrug.mfds.go.kr/kfda2

[25] US Food and Drug Administration. FDA Online label repository [Internet]. [cited 8 Jan 2016]. http://labels.fda.gov//

[26] Rehan HS, Chopra D, Sah RK, Mishra R. Adverse drug reactions: trends in a tertiary care hospital. Curr Drug Saf. 2012;7: 384–8. Available from: http://www.ncbi.nlm.nih.gov/pubmed/23150923 23150923

[27] Lobo MGA de A, Pinheiro SMB, Castro JGD, Momenté VG, Pranchevicius M-CS. Adverse drug reaction monitoring: support for pharmacovigilance at a tertiary care hospital in Northern Brazil. BMC Pharmacol Toxicol. 2013;14: 5 10.1186/2050-6511-14-5 23298396 PMC3554560

[28] Patidar D, Rajput MS, Nirmal NP, Savitri W. Implementation and evaluation of adverse drug reaction monitoring system in a tertiary care teaching hospital in Mumbai, India. Interdiscip Toxicol. 2013;6: 41–6. 10.2478/intox-2013-0008 24170978 PMC3795320

[29] Aagaard L, Weber CB, Hansen EH. Adverse drug reactions in the paediatric population in Denmark: a retrospective analysis of reports made to the Danish Medicines Agency from 1998 to 2007. Drug Saf. 2010;33: 327–39. 10.2165/11319100-000000000-00000 20297864

[30] Kim H, Seong J, Yang BR, Jin X, Choi N, Lee J, et al Analysis of Adverse Events Reporting Patterns and Signal Detection for Pediatric Patients in the Korean Spontaneous Reporting Data. J Pharmacoepidemiol risk Manag. 2012;5: 40–5.

[31] Star K, Norén GN, Nordin K, Edwards IR. Suspected adverse drug reactions reported for children worldwide: an exploratory study using VigiBase. Drug Saf. 2011;34: 415–28. 10.2165/11587540-000000000-00000 21513364

[32] Dang A, Bhandare PN. The profile of voluntary reported adverse drug reactions at a tertiary care hospital: a fifteen month prospective study. J Clin Diagn Res. 2012;6: 1504–9. 10.7860/JCDR/2012/4340.2544 23285441 PMC3527781

[33] Gor A, Desai S. Adverse Drug Reactions (ADR) in the inPatients of Medicine Department of a Rural Tertiary Care Teaching Hospital and Influence of Pharmacovigilance in Reporting ADR. Indian J Pharmacol. 2008;40: 37 10.4103/0253-7613.40488 21264160 PMC3023121

[34] Haile DB, Ayen WY, Tiwari P. Prevalence and assessment of factors contributing to adverse drug reactions in wards of a tertiary care hospital, India. Ethiop J Health Sci. 2013;23: 39–48. Available from: http://www.pubmedcentral.nih.gov/articlerender.fcgi?artid=3613814&tool=pmcentrez&rendertype=abstract 23559837 PMC3613814

[35] Ilhan MN, Durukan E, Ilhan SO, Aksakal FN, Ozkan S, Bumin MA. Self-medication with antibiotics: questionnaire survey among primary care center attendants. Pharmacoepidemiol Drug Saf. 2009;18: 1150–1157. 10.1002/pds.1829 19827009

[36] Kim JM, Lee YM. Trends of Adverse Drug Reactions (ADRs) -Related Admissions and Hospital Acquired ADRs in a Korean Tertiary Hospital. J Kor Soc Heal Pharm. 2015;32: 318–334.

[37] Eileen Kairuz T, Gargiulo D, Bunt C, Garg S. Quality, Safety and Efficacy in the “Off-Label” Use of Medicines. Curr Drug Saf. 2007;2: 89–95. 18690954 10.2174/157488607779315471

[38] Valente S, Murray L, Fisher D. Nurses improve medication safety with medication allergy and adverse drug reports. J Nurs Care Qual. 2007;22: 322–7. 10.1097/01.NCQ.0000290413.04522.0b 17873729

[39] Bergqvist M, Ulfvarson J, Andersen Karlsson E, von Bahr C. A nurse-led intervention for identification of drug-related problems. Eur J Clin Pharmacol. 2008;64: 451–456. 10.1007/s00228-007-0449-3 18204835

[40] van Puijenbroek EP, Bate A, Leufkens HG, Lindquist M, Orre R, Egberts AC. A comparison of measures of disproportionality for signal detection in spontaneous reporting systems for adverse drug reactions. Pharmacoepidemiol Drug Saf. 2002;11: 3–10. 10.1002/pds.668 11998548

